# Supplementary material for: Dostarlimab or pembrolizumab plus chemotherapy in previously untreated metastatic non-squamous non-small cell lung cancer: the randomized PERLA phase II trial
Source: Nat Commun. 2023 Nov 11;14:7301. doi: 10.1038/s41467-023-42900-4 (PMC10640551; doi:10.1038/s41467-023-42900-4)
Supplement: Supplementary file 1 — Supplementary Information [file 41467_2023_42900_MOESM1_ESM.pdf]

Supplementary Information

Supplementary Results

Results of primary analyses

Overall Response Rate (ORR)

At data cut-off of August 4, 2022, patients in the dostarlimab plus chemotherapy group received a mean of 10.1 cycles of dostarlimab (standard deviation: 6.0) and 9.3 cycles of pemetrexed (standard deviation: 5.7), with 79% of patients completing all 4 cycles of platinum-based chemotherapy. Patients in the pembrolizumab plus chemotherapy group received a mean of 8.3 cycles of pembrolizumab (SD: 5.7) and 8.0 cycles of pemetrexed (SD: 5.6), with 72% of patients completing all 4 cycles of platinum-based chemotherapy. For dostarlimab plus chemotherapy, the confirmed ORR per Response Evaluation Criteria in Solid Tumors (RECIST) version (v) 1.1 based on blinded independent central review (BICR) was 46% (56/121; 95% confidence interval [CI]: 37–56%), with two complete responses (CRs) (2%) and 54 partial responses (PRs) (45%). For pembrolizumab plus chemotherapy, ORR was 37% (45/122; 95% CI: 28–46%), with three CRs (2%) and 42 PRs (34%) (9% difference [80% CI: 1.46–17.18%; 95% CI: -2.70–21.33%]) (**Supplementary Table 5**). BICR-assessed maximum percentage reduction from baseline in tumor measurement is shown in **Supplementary Figure 2**. Five patients (4%) in the dostarlimab treatment group and 13 patients (11%) in the pembrolizumab treatment group had unknown or missing responses and were classified as 'not done' (**Supplementary Table 4; Supplementary Table 5**). These results are supported by the analysis of ORR based on Investigator assessment, which was 41% (50/121; 95% CI: 32–51%) in the dostarlimab plus chemotherapy treatment group and 29% (35/122; 95% CI: 21–38%) in the pembrolizumab plus chemotherapy group.

Subgroup analyses of ORR based on BICR by PD-L1 status are shown in **Supplementary Table 5** and **Supplementary Table 6**. Differences in ORR between treatment groups by PD-L1 status are shown in **Supplementary Figure 3**. The highest ORR was observed in the tumor proportion score (TPS) ≥50% subgroups of both dostarlimab plus chemotherapy (74% [20/27; 95% CI: 54–89%]) and pembrolizumab plus chemotherapy treatment groups (48% [13/27; 95% CI: 29–

68%]). Patients in the PD-L1-positive (TPS ≥1%) subgroup treated with dostarlimab plus chemotherapy had an ORR of 59% (42/71; 95% CI: 47–71%), while those treated with pembrolizumab plus chemotherapy had an ORR of 39% (28/71; 95% CI: 28–52%). ORR for PD-L1-negative (TPS <1%) patients was 28% (14/50; 95% CI: 16–43%) in the dostarlimab plus chemotherapy subgroup and 33% (17/51; 95% CI: 21–48%) in the pembrolizumab plus chemotherapy subgroup.

Safety

Overall, the safety profiles of dostarlimab plus chemotherapy and pembrolizumab plus chemotherapy were similar (**Supplementary Table 10**; **Supplementary Figure 5**). The proportion of patients experiencing any treatment-emergent adverse event (TEAE) was the same for both treatment groups (97%). The most frequent TEAEs were largely balanced between the two groups, although a smaller proportion of patients in the dostarlimab plus chemotherapy group experienced neutropenia than in the pembrolizumab plus chemotherapy group (12% vs 22%, respectively) (**Supplementary Table 11**; this difference diminished over time (17% vs 22%, respectively; data cut-off July 7, 2023). The proportion of patients experiencing any treatment-related adverse event (TRAE) was similar between groups (82% for dostarlimab plus chemotherapy and 79% for pembrolizumab plus chemotherapy). The four most frequent TRAEs (related to any study treatment) in both the dostarlimab plus chemotherapy and pembrolizumab plus chemotherapy groups were anemia (37% and 38%, respectively), asthenia (18% and 22%, respectively), nausea (18% and 19%, respectively), and neutropenia (12% and 18%, respectively) (**Supplementary Table 11**). The most frequent TRAEs related to dostarlimab or pembrolizumab specifically were anemia and rash (10% each) in the dostarlimab plus chemotherapy group and asthenia (13%) in the pembrolizumab plus chemotherapy group (**Supplementary Table 11**).

While more patients experienced dostarlimab-related AEs (64%) than pembrolizumab-related AEs (53%), a numerical trend favoring dostarlimab was observed in the proportion of patients experiencing immune-related adverse events (AEs) (26% for dostarlimab plus chemotherapy and 34% for pembrolizumab plus chemotherapy), serious adverse events (SAEs) (38% and 45%,

respectively), AEs leading to treatment discontinuation (25% and 32%, respectively), and AEs leading to immunotherapy discontinuation (15% and 24%, respectively) (**Supplementary Table 10**). Fatal TRAEs were observed in 2% of patients in the dostarlimab plus chemotherapy group and 4% of patients in the pembrolizumab plus chemotherapy group; individual fatal TRAEs are also summarized in **Supplementary Table 10**.

Supplementary Table 1. Institutions and IEC/IRB committees

Institution	IEC/IRB Committee
Clinica Universitaria Reina Fabiola	CIEIS Fabiola
Instituto de Investigaciones Metabólicas	CEIC Dr. Stamboulian
Fundacion Respirar	CIEFC- CIE para Ensayos en Farmacología Clínica
Centro Oncológico Riojano Integral	CEIC Dr. Stamboulian
Centro de Investigacion Pergamino*	Comité de Ética en Investigación Fundación OncoSalud
Clinica Viedma S.A.	Comité de Ética en Investigación Clínica (CEIC)
CEMER	Comite de etica en Investigacion CEMER
Centro Polivalente de Asistencia e Inv. Clinica CER	CEIC Dr. Stamboulian
Centro Oncologico de Rosario	Instituto de Oncología y Especialidades Médicas
Centro Platense en Investigaciones Respiratorias	Comité de Ética Instituto Médico Platense
Núcleo de Pesquisa Clínica da Rede São Camilo	Comitê de Ética em Pesquisa do Instituto Brasileiro de Controle do Câncer
Hospital Santa Rita, Centro de Pesquisa Clínica	Hospital Univ Cassiano Antônio de Moraes da Univ Federal do Espírito Santo
CRIO - Centro Regional Integrado de Oncologia	Comitê de Ética em Pesquisa da Universidade Federal do Caerá
INCA - Instituto Nacional do Cancer	Comitê de Ética em Pesquisa - CEP – INCA
Sociedade Beneficência e Caridade de Lajeado – Hospital Bruno Born*	Comite de Etica em Pesquisa da Universidade do Vale do Taquari – UNIVATES
Liga Norte RioGrandense Contra o Câncer	Com de Et em Pesq com Seres Humanos da Liga Norte Riograndense Cont o Canc
Fundação PIO XII - Hospital de Câncer de Barretos	Comite de Etica em Pesquisa da Fundacao Pio XII
Orlandi Oncologia	Comité de Ética Científico SSMO
Centro de Investigaciones del Cancer James Lind*	NA[†]
Centro de estudios Clinicos SAGA Spa*	Comité de Ética Científico SSMO

CHU de Caen - Hôpital Côte de Nacre	CPP Ile-de-France II - Hôpital Necker
Institut de Cancérologie de l'Ouest - Site Saint Herblain	CPP Ile-de-France II - Hôpital Necker
Clinique Teissier – Groupe AHNAC	CPP Ile-de-France II - Hôpital Necker
CH Le Mans, Pneumologie – Oncologie Thoracique	CPP Ile-de-France II - Hôpital Necker
CHU de Bordeaux - GH Sud - Hôpital Haut-Lévêque	CPP Ile-de-France II - Hôpital Necker
CHU de Limoges - CHU Dupuytren 1	CPP Ile-de-France II - Hôpital Necker
Krankenhaus Merheim	Ethikkommission der Aerztekammer Nordrhein
Krankenhaus Nordwest	Ethik-Kommission der Landesärztekammer Hessen
Pius Hospital	Carl von Ossietzky Univerität Oldenburg, Medizinische Ethikkommission
St. Vincentius-Kliniken gAG Karlsruhe*	NA[†]
Charite-Universitaetsmedizin Berlin	Ethikkommission des Landes Berlin
Fachklinik f. Lungenerkrankungen	Ethik-Kommission der Landesärztekammer Hessen
Studienzentrum Haematologie/Onkologie/Diabetologie*	Ethik-Kommission der Bayerischen Landesärztekammer
Klinikum Kassel GmbH*	Ethik-Kommission der Landesärztekammer Hessen
Arnas Garibaldi – P.O. Nesima	Segreteria Scientifico-Amministrativa
Centro Di Riferimento Oncologico – IRCCS – Sevizio Sanitario Regionale FVG	Comitato Etico Regionale Unico c/o AOU Santa Maria della Misericordia
Istituto Europeo di Oncologia	Com.Etico IRCCS – Ist. Europeo di Oncologia e Centro Cardiologico Monzino
Spedali Civili di Brescia	Comitato Etico di Brescia
Istituto Nazionale dei Tumori IRCCS	Comitato Etico Ind dell'Istutituto Naz per lo Studio e la Cura dei Tumori
Azienda Ospedaliera San Camillo Forlanini	Comitato Etico Lazio 1, Segreteria Scientifico-Amministrativa
A.O.R.N. Ospedali dei Colli "Monaldi-Cotugno-CTO	Comitato Etico dell'A.O.R.N. dei Colli di Napoli
Samsung Medical Center	Samsung Medical Center
Asan Medical Center	Asan Medical Center

Severance Hospital	Severance Hospital
Chungbuk National University Hospital	Chungbuk National University Hospital
Inje University Haeundae Paik Hospital	Inje University Haeundae Paik Hospital
Centrum Medyczne Pratia*	Kom Bioet przy Okręgowej Izbie Lekarskiej Wielkopolskiej Izby Lekarskiej
Samodzielny Publiczny Zespol Gruzlicy I Chorob Pluc	Kom Bioet przy Okręgowej Izbie Lekarskiej Wielkopolskiej Izby Lekarskiej
Samodzielny Publiczny Szpital Kliniczny w Lublinie	Kom Bioet przy Okręgowej Izbie Lekarskiej Wielkopolskiej Izby Lekarskiej
Med-Polonia Sp. z o. o. NSZOZ	Kom Bioet przy Okręgowej Izbie Lekarskiej Wielkopolskiej Izby Lekarskiej
Ars Medical Sp. z o.o*	Kom Bioet przy Okręgowej Izbie Lekarskiej Wielkopolskiej Izby Lekarskiej
Centrum Terapii Wspolczesnej	Kom Bioet przy Okręgowej Izbie Lekarskiej Wielkopolskiej Izby Lekarskiej
Centrum Onkologii im. prof. F. Lukaszczyka	Kom Bioet przy Okręgowej Izbie Lekarskiej Wielkopolskiej Izby Lekarskiej
SC Radiotherapy Center Cluj SRL	Comisia Nationala de Bioetica a medicamentelor si a Dispozitivelor Medicale
Spitalul de Psihiatrie Titan Dr Constantin Gorgos	National Ethics committee
Centrul de Oncologie "Sf. Nectarie"	Comisia Nationala de Bioetica a medicamentelor si a Dispozitivelor Medicale
Hospital del Mar	Hospital del Mar
Complejo Hospitario Nuestra	Hospital del Mar
Complejo Hospitalario de Jaén	Hospital del Mar
Hospital Universitario Virgen de la Victoria	Hospital del Mar
Hospital Universitario Lucus Augusti	Hospital del Mar
Tri-Service General Hospital	Tri-Service General Hospital
Changhua Christian Hospital	Changhua Christian Hospital
The Texas Cancer Center*	United States Oncology Incorporated Institutional Review Board
Rocky Mountain Cancer Centers-Sky Ridge	United States Oncology Incorporated Institutional Review Board

Cancer Care Centers of Brevard, Incorporated*	United States Oncology Incorporated Institutional Review Board
Virginia Cancer Specialists	United States Oncology Incorporated Institutional Review Board
Woodlands Medical Specialists*	United States Oncology Incorporated Institutional Review Board
Texas Oncology - Tyler*	United States Oncology Incorporated Institutional Review Board
Oncology Hematology Care, Incorporated	United States Oncology Incorporated Institutional Review Board

*No patients enrolled; [†]study site not activated.

NA, not applicable.

Supplementary Table 2. Protocol deviations

**Description of change**	**Brief rationale**
Minor editorial changes as needed to align with Sponsor's protocol template	Alignment with the Sponsor's standard protocol template and ways of working
Included *"BRAF V600E mutations and other genomic aberrations for which an approved targeted therapy is available"* in the disease eligibility criterion	Per Health Authority feedback, these patients should not be included in the study as there are available targeted treatments for their disease
Removed IMP and NIMP definitions from table of study treatments administered	As definitions of IMP and NIMP of the chemotherapies administered in the protocol may vary across countries, these have been removed for clarity
Changed "*use*" listed for pembrolizumab in table of study treatments administered from "*background intervention*" to *"comparator"*	Pembrolizumab is being used as a comparator in this study
Schedule of activities amended to correct inaccurate infusion times for chemotherapy agents	Revision to correct an error in the study protocol
Updated efficacy and safety information for dostarlimab added to protocol introduction	Revision to incorporate updated information added at the request of Health Authority feedback
Reasons for required withdrawal/discontinuation was updated to more explicitly differentiate between scenarios requiring discontinuation and potential scenarios	Revision to address request of Health Authority
Instructions for emergency unblinding during the course of the study added	Revision to provide more comprehensive information consistent with the Blinding Plans
Instructions for dose modifications updated in tables of dose levels and dose adjustment	Revisions to ensure that information is consistent with the product information for the study interventions utilized in the study

Clarification added that PK and immunogenicity for pembrolizumab will be assessed only if needed	Dostarlimab PK and immunogenicity samples analyzed as scheduled, but pembrolizumab samples analyzed only if the study Sponsor determines necessary. The population for immunogenicity analysis includes a broader population to enable further subset analysis, including the one currently defined.
Correction to timeframe for reporting AEs. Rather than all AEs being reported from the signing of informed consent, only TRSAEs will be reported from the signing of informed consent and all AEs will be reported from the start of study treatment	Alignment of the protocol language throughout with GSK guidance on AE/SAE collection
Correction of timing for baseline tumor assessment of the chest and abdomen from within 28 days prior to first dose to 35 days	Clarifying that the window for baseline tumor assessments should be 28 (+7 days), as opposed to 28 (+/- 7 days)
Text updated as below (updates in **bold**):<br>*"**The CT component of** positron emission tomography (PET)/CT may be used according to RECIST v1.1 guidelines, with full radiation dose diagnostic CT and IV CT contrast, and as clinically indicated"*	Clarifying that the CT component of diagnostic quality PET/CT may be used for tumor assessment per RECIST v1.1 guidelines; prior text indicated that PET/CT could be used for diagnostic quality
Text updated as below (updates in **bold**):<br>*"Treatment cycles are 21 days long with **dosing scheduled** on D1 of each cycle. **All assessment should be done prior to drug administration and within 72 h prior to 1st dose of study drugs administered in that cycle, unless otherwise specified (NB: this includes C1D1 and all cycles).** Visits **starting at Cycle 2 can be performed** ±3 days **of the scheduled date"***	Protocol updated to allow flexibility for sample collection at Day 1
For archival (or fresh) tumor tissue samples, additional instruction added:	Expanding the window when the sample can be sent for biomarker analysis to allow greater flexibility in obtaining a sample

"If PD-L1 status is known and the participant has agreed to provide archival sample via the optional consent, this sample may be provided within 28 days of the start of study treatment"	
Additional assessment "*Evaluate genomic aberration*" added to schedule of activities table	Indicated in the schedule of assessment that results of genomic aberrations are needed at screening. This was to align with the rest of the protocol
Additional instruction added to the schedule of activities of table for optional blood sample for genetic testing to clarify sample may be obtained after randomization during first cycle if not collected at screening	Allowing flexibility for sample collection to reduce collection burden at Day 1
PRO collection timepoints updated in schedule of activities table to reflect timepoints in cycles rather than weeks	Added to align the protocol with other study documents
Updated schedule of activities table to reflect that PROs should be collected prior to any clinical procedures, whenever possible	Allowing flexibility for these assessments, reducing both burden to participants and the operational burden to site staff
Clarification added to schedule of activities table that urinalysis starts at Cycle 6, Day 1	Clarification
Clarification added to schedule of activities table that urinalysis starts at Cycle 2, Day 1	Clarification
Removed statement on EU approval of pembrolizumab monotherapy in NSCLC	Corrected an error as pembrolizumab is only approved for the indication described in the introduction in the US
Added additional detail on the potential risks of exposure to ionizing radiation due to the inclusion in the study of CT scans	Added to include risk assessment considerations related to radiographic imaging and radiation exposure

Protocol revised to note the duration of contraception for female and males post-chemotherapy must be consistent with local regulations with a minimum of 180 days for males and females, consistent with dostarlimab guidance	Revision to incorporate additional flexibility as may be guided by local labelling requirements
Reworded inclusion criterion (updates in strikethrough and **bold**):<br><br>~~Target~~ **Measurable** lesions situated in a previously irradiated area ~~are~~ **may be** considered ~~measurable~~ **target** lesions in progression has been demonstrated in such lesions and if there are other target lesions	Clarification
Reworded exclusion criterion (updates in strikethrough and **bold**):<br><br>Participant has an additional malignancy or a history of prior malignancy, with the exception of adequately treated basal or squamous skin cancer, cervical carcinoma in situ, ~~or~~ **superficial** bladder ~~carcinoma in situ~~ **cancer** without evidence of disease, **other in situ cancers**, or had a malignancy treated with curative intent and with no evidence of disease recurrence for 5 years since the initiation of that therapy.	Inconsistency identified during study conduct. Previously allowed one type of superficial bladder cancer but not others of similar or lesser risk.
Removed the requirement for participants who test positive for the presence of hepatitis B core antibody to be excluded	Included in error and not applicable to this study
The protocol was amended to provide additional flexibility for local guidelines with relation to infusion times for chemotherapy agents	Revision to correct discrepancy in the study protocol introduced by way of regional differences in labelling instructions
Instructions for the collection of PROs updated	To correct an error in the collection method for PROs when subjects are unable to attend a clinic visit
Added definition of best overall response as **confirmed** CR or PR	Update to clarify that response confirmation is required for ORR analysis

Added that samples may be used for additional exploratory biomarker testing and for the development of a diagnostic test	Additional language to ensure that the information is aligned with elsewhere in protocol, the exploratory biomarker objectives and potential future use for CDx development
"*Future Biomedical Research*" section renamed to *"Biomedical Research"*	This section contains both planned analysis for the current study and potential future investigations
Additional text added to describe a potential, future population PK analysis including data from this study	Population PK analysis will be performed if study sponsor determines it is necessary
Amended the use of X-rays for the assessment of measurable lesions to the assessment of progression due to new lesions	Clarification: CT/MRI should be used to assess measurable/target lesions; X-rays are not appropriate for target lesion assessment in this study
Removed the definition of a measurable lesion as ≥10 mm caliper/ruler measurement by clinical examination or medical photography	Clarification: CT/MRI should be used to assess measurable/target lesions; clinical exam and photography are not appropriate to use for target lesion assessment in this study
Added statement to clarify laboratory assessments for each cycle can occur up to 72 hours before treatment	Alleviating patient burden by reducing time at site on day 1
Removed all references to the eCRF requirement for documenting causality review	Alignment with current protocol template guidance
Corrected typographical error incorrectly capturing GARNET lung cancer population as previously "*untreated*" NSCLC patients	Typographical error
Criteria for treatment withdrawal/stopping rules was clarified to include the text *"or other adverse event"* to the criterion of unacceptable toxicity	Additional editorial revisions to provide further clarity to the study design

Text updated (shown in **bold**):<br><br>*"Tumor tissue will be evaluated for PD-L1 expression using IHC and may be correlated with ORR* ***and potentially other clinical endpoints*** *to treatment"*	To expand the potential scope of this exploratory endpoint
Pemetrexed should be administered according to the local label, thus guidance on infusion rate has been removed	In response to local requirements, sites are allowed to follow their local recommendations
Instructions for dose modifications for non-hematological chemotherapy toxicities updated. Removed the requirement for PD-1 inhibitors to be withheld in the event of all drug-related toxicities. Instructions for dose modification guidelines for PD-1 inhibitor-related AEs updated	Revisions to ensure that information is consistent with the product information for the study interventions utilized in the study
Removed number of participants limit per strata	Due to the standard of care treatment available to participants whose TPS score is >50%, enrolment into this stratum is relatively limited. Therefore, by removing the requirement to evenly distribute participants across three strata, it is anticipated that study enrolment rate and accessibility will benefit
Post-dosing timepoints for PK and biomarker sample collection is done after immunotherapy infusion	Clarified that post-dose collection timepoints refer to immunotherapy dosing and not patient treatment, including chemotherapy
"Permanently discontinue" for Grade 4 severity was removed from adrenal insufficiency, hypophysitis, hypothyroidism and hyperthyroidism	Clinical management of these endocrinopathies are amendable to hormone replacement therapies or thyroid suppressive therapy and the adequacy of supplementation monitorable symptomatically and with laboratory assessments

Updates to dose modifications:<br>• Updated dose modifications for adrenal insufficiency to include endocrine consultation as follow-up<br>• Expanded dose modifications for Guillain-Barré syndrome to include several severe neurological events (myasthenic syndrome/myasthenia gravis and transverse myelitis were added), to include Grade 2, to include corticosteroid and other therapy guidelines, and to include neurology consultation as follow-up<br>• Updated dose modifications for myocarditis<br>• Added DRESS to dose modifications for rash/skin reactions<br>• Added corticosteroid and other therapy guidelines and consultation follow-up for other irARs	Clarification/correction/program updates, including mitigation of potentially serious complications of immune-related events, including severe neurological events, severe skin reactions and myocarditis
Removed footnote that decision to withhold or discontinue PD-1 inhibitor is at the discretion of the investigator or treating physician	Incorrect footnote removed to ensure clarity about the reason to discontinue or withhold PD-1 inhibitor. This decision should be deduced from the protocol instructions rather than based on investigator assessment.

(N)IMP, (non-)investigational medicinal product; AE, adverse event; C, cycle; CDx, clinical diagnostics; CR, complete response; CT, computed tomography; D, day; DRESS, drug reaction with eosinophilia and systemic symptoms; eCRF, electronic case report form; h, hour; irAR, immune-related adverse reaction; IV, intravenous; MRI, magnetic resonance imaging; ORR, overall response rate; PD-(L)1, programmed death (ligand) 1; PET, positron emission tomography; PK, pharmacokinetics; PR, partial response; PRO, patient-reported outcome; RECIST, Response Evaluation Criteria in Solid Tumors; SAE, serious adverse event; TRSAE, treatment-related serious adverse event; v, version.

Supplementary Table 3. Full inclusion and exclusion criteria

Inclusion criteria	Exclusion criteria
• ≥18 years old, able to understand study procedures, and agrees to participate in the study by providing written informed consent, which includes compliance with the requirements and restrictions listed in the ICF and in the study protocol<br>• Histologically- or cytologically- confirmed metastatic non-squamous NSCLC with documented absence of a sensitizing EGFR, ALK, ROS-1, or BRAF V600E mutation or other genomic aberration for which an approved targeted therapy is available; mixed tumors will be categorized by the predominant cell type; if the tumor has predominantly squamous cell histology or if small cell elements are present, participant is ineligible<br>• Measurable disease (presenting with at least 1 measurable lesion per RECIST v1.1 as determined by the local site investigator/radiology assessment); measurable lesions in previously irradiated areas may be considered target lesions if progression has been demonstrated in such lesions and if there are other target lesions (ineligible if only 1 target lesion previously irradiated)<br>• Documented PD-L1 status by the 22C3 pharmDx assay<br>• Eastern Cooperative Oncology Group (ECOG) performance status score of 0 or 1<br>• Life expectancy ≥3 months	• Prior systemic therapy for the treatment of metastatic NSCLC; participants who received neoadjuvant or adjuvant chemotherapy are eligible if completed ≥12 months prior to development metastatic disease<br>• Prior therapy with a PD-(L)1/2 inhibitor, CTLA-4 inhibitor, TIM-3 inhibitor, or any other IO agent for the treatment of cancer<br>• Radiation to the lung >30 Gy within 6 months of the first dose of study treatment<br>• Completion of palliative radiotherapy within 7 days of the first dose of study treatment<br>• Any of the following hepatic characteristics present:<br>○ ALT >2.5x upper limit of normal (ULN) without liver metastases/tumor infiltration<br>○ ALT >5x ULN with liver metastases/tumor infiltration<br>○ Bilirubin >1.5x ULN (isolated bilirubin >1.5x ULN acceptable if bilirubin is fractionated and direct bilirubin <35%)<br>○ Current active liver or biliary disease (exceptions: Gilbert's syndrome, asymptomatic gallstones, liver metastases, otherwise stable chronic liver disease per Investigator assessment)<br>• QTc >450 ms (>480 ms for participants with BBB)<br>• Major surgery within 3 weeks of the first dose of study treatment or has not adequately recovered from any AEs (Grade ≤1) and/or

• Adequate organ function (hematologic, hepatic, renal)<br>• Recovered to Grade ≤1 from any prior treatment-related toxicities at the time of randomization (exception: Grade 2 alopecia)<br>• Contraceptive use consistent with local regulations regarding methods of contraception for those participating in clinical studies<br>• Male participants must agree to the following for the treatment period and for ≥180 days after the last dose of study treatment:<br>○ Refrain from donating sperm<br>○ Either be abstinent from heterosexual intercourse as their preferred and usual lifestyle and agree to remain abstinent OR agree to use a male condom when having sexual intercourse with a woman of childbearing potential who is not currently pregnant or when engaging in any activity that allows for passage of ejaculate to another person<br>• Female participants must not be pregnant or breastfeeding and one of the following conditions must apply:<br>○ Be of non-childbearing potential<br>○ Be of childbearing potential but use a highly effective contraceptive method (failure rate <1% per year, preferably with low user dependency) during the treatment period and for ≥180 days after the last dose and agree not to donate eggs for the purposes of reproduction during the same period; participants of child-bearing potential should have a negative highly sensitive	complications from major surgery; surgical implantation of a port catheter is not exclusionary<br>• Additional malignancy or a history of prior malignancy (exceptions: adequately treated basal or squamous skin cancer, cervical carcinoma in situ, superficial bladder cancer without evidence of disease, other in situ cancers, malignancy treated with curative intent and with no evidence of disease recurrence for 5 years since the initiation of that therapy)<br>• Known active brain metastases and/or leptomeningeal metastases (exceptions: participants who have received prior therapy for their brain metastases and have radiographically stable central nervous system disease and are neurologically stable for at least 2 weeks before study entry and off corticosteroids within 3 days prior to the first dose of study treatment, or participants with known untreated, asymptomatic brain metastases). Exception: participants with known untreated asymptomatic brain metastases (i.e., no neurological symptoms, requirements for corticosteroids, lesions >1.5 cm, and no or minimal surrounding edema)<br>• Positive test for hep B surface antigen or positive hep C antibody test result at screening/within 3 months prior to first dose of study treatment<br>• Active infection requiring systemic therapy within 1 week prior to the anticipated first dose of study treatment<br>• Known HIV

pregnancy test within 72 hours before the first dose of study treatment	• Active autoimmune disease that required systemic treatment in the past 2 years, is immunocompromised in the opinion of the investigator, or is receiving systemic immunosuppressive treatment (replacement therapy not considered a systemic treatment)<br>• Received systemic steroid therapy within 3 days prior to the first dose of study treatment or receiving any other form of immunosuppressive medication (inhaled corticosteroids, local steroid injections, or steroid eye drops is allowed)<br>• Symptomatic ascites or pleural effusion (exception: clinically stable following treatment of these conditions)<br>• Current interstitial lung disease, current pneumonitis, or a history of pneumonitis that required the use of oral or IV glucocorticoids to assist with management<br>• History or current evidence of any medical condition, therapy, or laboratory abnormality that might confound the study results, interfere with their participation for the full duration of the study treatment, or indicate it is not in the best interest of the participant to participate, in the opinion of the Investigator<br>• Clinically active diverticulitis, intra-abdominal abscess, gastrointestinal obstruction, or peritoneal carcinomatosis<br>• Pre-existing peripheral neuropathy that is Grade ≥2 by National Cancer Institute-Common Terminology Criteria for Adverse Events (NCI-CTCAE) v5.0 criteria<br>• Live vaccine within 30 days of the first dose of study treatment

	• Not meeting requirements per local prescribing guidelines for receiving treatment with either pemetrexed and cisplatin or carboplatin<br>• Sensitivity to any of the study treatments, or components thereof, or a history of drug or other allergy that, in the opinion of the investigator or GSK Medical Monitor, contraindicates their participation<br>• Unable to interrupt aspirin or other nonsteroidal anti-inflammatory drugs (NSAIDs), other than an aspirin dose ≤1.3 g per day, for a 5-day period (8-day period for long-acting agents, such as piroxicam)

AE, adverse event; ALT, alanine transaminase; BBB, bundle branch block; CTLA-4, cytotoxic T-lymphocyte associated protein 4; HIV, human immunodeficiency virus; IO, immunotherapy; NCI-CTCAE, National Cancer Institute-Common Terminology Criteria for Adverse Events; NSAIDs, non-steroidal anti-inflammatory drugs; NSCLC, non-small cell lung cancer; PD-(L)1/2, programmed death (ligand) 1/2; QTc, corrected QT interval; RECIST v1.1, Response Evaluation Criteria in Solid Tumors version 1.1; TIM-3, T-cell immunoglobulin and mucin domain 3; ULN, upper limit of normal.

Supplementary Table 4. Reasons for unknown or missing ('not done') patient responses

Reasons for unknown or missing responses	Dostarlimab + CT (n=5)	Pembrolizumab + CT (n=13)
**No post-baseline disease assessments available due to death from a fatal AE**	2 (40)	8 (62)
**No post-baseline disease assessments available due to death from disease under study**	3 (60)	1 (8)
**No post-baseline disease assessments available due to withdrawal from study***	0 (0)	2 (15)
**Started new anti-cancer therapy before first assessment**	0 (0)	1 (8)
**Independent BICR assessment missing**	0 (0)	1 (8)

*Withdrawal reasons were withdrawal of consent (n=1) and went into hospice care (n=1). Responses are applicable to both the primary and updated analyses.

AE, adverse events; BICR, blinded independent central review; CT, chemotherapy.

Supplementary Table 5. ORRs for patients receiving dostarlimab + chemotherapy and pembrolizumab + chemotherapy (ITT population as of August 4, 2022)

	Dostarlimab + chemotherapy (N=121)	Pembrolizumab + chemotherapy (N=122)
**Best overall response, n (%)**		
Complete response	2 (2)	3 (2)
Partial response	54 (45)	42 (34)
Stable disease	48 (40)	52 (43)
Progressive disease	12 (10)	11 (9)
Not evaluable	0	1 (<1)
Not done*	5 (4)	13 (11)
**ORR**		
Complete response + partial response, n (%)	56 (46)	45 (37)
95% CI	37.2–55.6	28.3–46.1
**ORR by PD-L1 TPS subgroup, n/N (%)**		
TPS <1%	14/50 (28)	17/51 (33)
95% CI	16–43	21–48
TPS ≥1%	42/71 (59)	28/71 (39)
95% CI	47–71	28–52
TPS 1–49%	22/44 (50)	15/44 (34)
95% CI	35–65	21–50
TPS ≥50%	20/27 (74)	13/27 (48)
95% CI	54–89	29–68

ORR was assessed per RECIST v1.1 based on BICR.

*Patients with ORR listed as “not done” had unknown or missing responses. The reasons for unknown or missing responses in each arm are listed in **Supplementary Table 4**.

BICR, blinded independent central review; CI, confidence interval; ITT, intention-to-treat; ORR, overall response rate; PD-L1, programmed death ligand-1; RECIST v1.1, Response Evaluation Criteria in Solid Tumors version 1.1; TPS, tumor proportion score.

Supplementary Table 6. Summary of best overall responses by PD-L1 TPS subgroup for the primary analyses (ITT population as of August 4, 2022)

	Dostarlimab + chemotherapy (N=121)	Pembrolizumab + chemotherapy (N=122)
**TPS <1%, n (%)**		
Complete response	2 (4)	1 (2)
Partial response	12 (24)	16 (31)
Stable disease	29 (58)	23 (45)
Progressive disease	6 (12)	5 (10)
Not evaluable	0 (0)	0 (0)
Not done*	1 (2)	6 (12)
**TPS ≥1%, n (%)**		
Complete response	0 (0)	2 (3)
Partial response	42 (59)	26 (37)
Stable disease	19 (27)	29 (41)
Progressive disease	6 (8)	6 (8)
Not evaluable	0 (0)	1 (1)
Not done*	4 (6)	7 (10)
**TPS 1–49%, n (%)**		
Complete response	0 (0)	0 (0)
Partial response	22 (50)	15 (34)
Stable disease	15 (34)	17 (39)
Progressive disease	5 (11)	6 (14)
Not evaluable	0 (0)	1 (2)
Not done*	2 (5)	5 (11)
**TPS ≥50%, n (%)**		
Complete response	0 (0)	2 (7)
Partial response	20 (74)	11 (41)
Stable disease	4 (15)	12 (44)
Progressive disease	1 (4)	0 (0)
Not evaluable	0 (0)	0 (0)
Not done*	2 (7)	2 (7)

ORR was assessed by BICR per RECIST v1.1. *Patients with ORR listed as “not done” had unknown or missing responses. The reasons for unknown or missing responses in each arm are listed in **Supplementary Table 2**.

BICR, blinded independent central review; ORR, overall response rate; PD-L1, programmed cell death ligand-1; RECIST v1.1, Response Evaluation Criteria in Solid Tumors version 1.1; TPS, tumor proportion score.

Supplementary Table 7. Median PFS by PD-L1 TPS subgroup for patients receiving dostarlimab + chemotherapy and pembrolizumab + chemotherapy (ITT population as of August 4, 2022)

	Dostarlimab + chemotherapy (N=121)	Pembrolizumab + chemotherapy (N=122)	HR (95% CI)
**TPS <1%**			0.77 (0.46–1.28)
N analyzed	50	51	
Median PFS, months (95% CI)	7.0 (4.9–9.7)	6.9 (4.7–9.6)	
**TPS ≥1%**			0.66 (0.41–1.03)
N analyzed	70	71	
Median PFS, months (95% CI)	10.4 (6.8–13.6)	6.1 (4.8–7.1)	
**TPS 1–49%**			0.67 (0.38–1.19)
N analyzed	44	44	
Median PFS, months (95% CI)	9.0 (5.3–NR)	5.4 (3.2–11.3)	
**TPS ≥50%**			0.60 (0.27–1.29)
N analyzed	27	27	
Median PFS, months (95% CI)	10.4 (5.8–NR)	6.7 (4.2–NR)	

PFS was assessed per RECIST v1.1 based on investigator assessment.

CI, confidence interval; HR, hazard ratio; ITT, intention-to-treat; NR, not reached; PD-L1, programmed death ligand 1; PFS, progression-free survival; RECIST v1.1, Response Evaluation Criteria in Solid Tumors version 1.1; TPS, tumor proportion score.

Supplementary Table 8. Primary OS analyses for patients receiving dostarlimab + chemotherapy and pembrolizumab + chemotherapy (ITT population as of August 4, 2022)

**Variable**	**Dostarlimab + chemotherapy (N=121)**	**Pembrolizumab + chemotherapy (N=122)**
Median OS follow-up time, months (IQR)	10.9 (7.7–13.2)	10.9 (7.5–13.3)
OS events observed, n	42	48
Median OS (95% CI), months	NR (12.6–NR)	14.0 (10.8–17.1)
Estimated probability of OS, % (95% CI)		
6 months	77 (68–83)	77 (69–84)
12 months	62 (51–71)	60 (50–69)

CI, confidence interval; IQR, interquartile range; ITT, intention-to-treat; NR, not reached; OS, overall survival.

Supplementary Table 9. Primary analyses of AEs by preferred term occurring in ≥10% of patients (safety population as of July 7, 2023)

AE (n, %)	Dostarlimab + chemotherapy (N=121)	Pembrolizumab + chemotherapy (N=122)
**TEAEs**	119 (98)	119 (98)
Alanine aminotransferase increased	15 (12)	15 (12)
Anemia	61 (50)	60 (49)
Aspartate aminotransferase increased	13 (11)	8 (7)
Asthenia	41 (34)	41 (34)
Back pain	8 (7)	12 (10)
Blood creatinine increased	16 (13)	9 (7)
Constipation	23 (19)	23 (19)
Cough	24 (20)	21 (17)
Decreased appetite	23 (19)	19 (16)
Diarrhea	21 (17)	18 (15)
Dyspnea	23 (19)	19 (16)
Fatigue	15 (12)	16 (13)
Nausea	30 (25)	31 (25)
Neutropenia	20 (17)	27 (22)
Peripheral oedema	13 (11)	11 (9)
Respiratory tract infection	8 (7)	13 (11)
Pneumonia	10 (8)	19 (16)
Pyrexia	14 (12)	17 (14)
Rash	15 (12)	7 (6)
Pruritus	11 (9)	14 (11)
Thrombocytopenia	14 (12)	11 (9)
Vomiting	24 (20)	15 (12)
**TRAEs***	103 (85)	99 (81)
Alanine aminotransferase increased	9 (7)	12 (10)
Anemia	50 (41)	48 (39)
Asthenia	25 (21)	28 (23)
Decreased appetite	12 (10)	13 (11)
Diarrhea	42 (35)	46 (38)
Nausea	24 (20)	24 (20)
Neutropenia	18 (15)	22 (18)

Rash	14 (12)	3 (2)
Thrombocytopenia	13 (11)	9 (7)
Vomiting	13 (11)	10 (8)
**TRAEs related to dostarlimab/pembrolizumab**	86 (71)	70 (57)
Anemia	15 (12)	9 (7)
Asthenia	11 (9)	17 (14)
Rash	13 (11)	3 (2)

*Adverse events described as treatment-related could be related to any study treatment agent.

AE, adverse event; TEAE, treatment-emergent adverse event; TRAE, treatment-related adverse event.

Supplementary Table 10. AE summary for patients receiving dostarlimab + chemotherapy and pembrolizumab + chemotherapy (safety population as of August 4, 2022)

AE, n (%)	Dostarlimab + chemotherapy (N=121)	Pembrolizumab + chemotherapy (N=122)
Any TEAE	117 (97)	118 (97)
TRAEs*	99 (82)	96 (79)
SAEs	46 (38)	55 (45)
TRSAEs*	20 (17)	29 (24)
irAEs	31 (26)	41 (34)
Grade ≥3 irAEs	11 (9)	17 (14)
irSAEs	9 (7)	11 (9)
AEs leading to any treatment discontinuation	30 (25)	39 (32)
AEs leading to dostarlimab/pembrolizumab discontinuation	18 (15)	29 (24)
Fatal TRAEs*	3 (2)	5 (4)
Immune-mediated lung disease	1 (<1)	0 (0)
Myelosuppression	0 (0)	1 (<1)
Pneumonia	0 (0)	1 (<1)
Pneumonitis	1 (<1)	0 (0)
Respiratory failure	0 (0)	1 (<1)
Septic shock	0 (0)	1 (<1)
Urosepsis	1 (<1)	0 (0)
Thrombocytopenia	0 (0)	1 (<1)

*AEs described as treatment-related could be related to any study treatment agent.

AE, adverse event; irAE, immune-related AE; SAE, serious adverse event; TEAE, treatment-emergent adverse event; TRAE, treatment-related adverse event; TRSAE, treatment-related SAE.

Supplementary Table 11. Primary analyses of AEs by preferred term occurring in ≥10% of patients (safety population as of August 4, 2022)

AE (n, %)	Dostarlimab + chemotherapy (N=121)	Pembrolizumab + chemotherapy (N=122)
**TEAEs**	117 (97)	118 (97)
Alanine aminotransferase increased	11 (9)	14 (11)
Anemia	54 (45)	59 (48)
Asthenia	39 (32)	39 (32)
Back pain	7 (6)	12 (11)
Blood creatinine increased	13 (11)	6 (5)
Constipation	23 (19)	21 (17)
Cough	22 (18)	20 (16)
Decreased appetite	18 (15)	15 (12)
Diarrhea	15 (12)	17 (14)
Dyspnea	21 (17)	18 (15)
Fatigue	14 (12)	12 (10)
Nausea	28 (23)	29 (24)
Neutropenia	15 (12)	27 (22)
Pneumonia	10 (8)	17 (14)
Pyrexia	12 (10)	15 (12)
Rash	14 (12)	6 (5)
Thrombocytopenia	13 (11)	10 (8)
Vomiting	20 (17)	14 (11)
**TRAEs***	99 (82)	96 (79)
Anemia	45 (37)	46 (38)
Asthenia	22 (18)	27 (22)
Decreased appetite	9 (7)	12 (10)
Nausea	22 (18)	23 (19)
Neutropenia	14 (12)	22 (18)
Rash	13 (11)	3 (2)
Thrombocytopenia	12 (10)	8 (7)
**TRAEs related to dostarlimab/pembrolizumab**	78 (64)	65 (53)
Anemia	12 (10)	8 (7)
Asthenia	10 (8)	16 (13)
Rash	12 (10)	3 (2)

*Adverse events described as treatment-related could be related to any study treatment agent.

AE, adverse event; TEAE, treatment-emergent adverse event; TRAE, treatment-related adverse event.

Supplementary Figure 1. DOR Kaplan–Meier curves for dostarlimab plus chemotherapy and pembrolizumab plus chemotherapy (ITT population as of July 7, 2023)

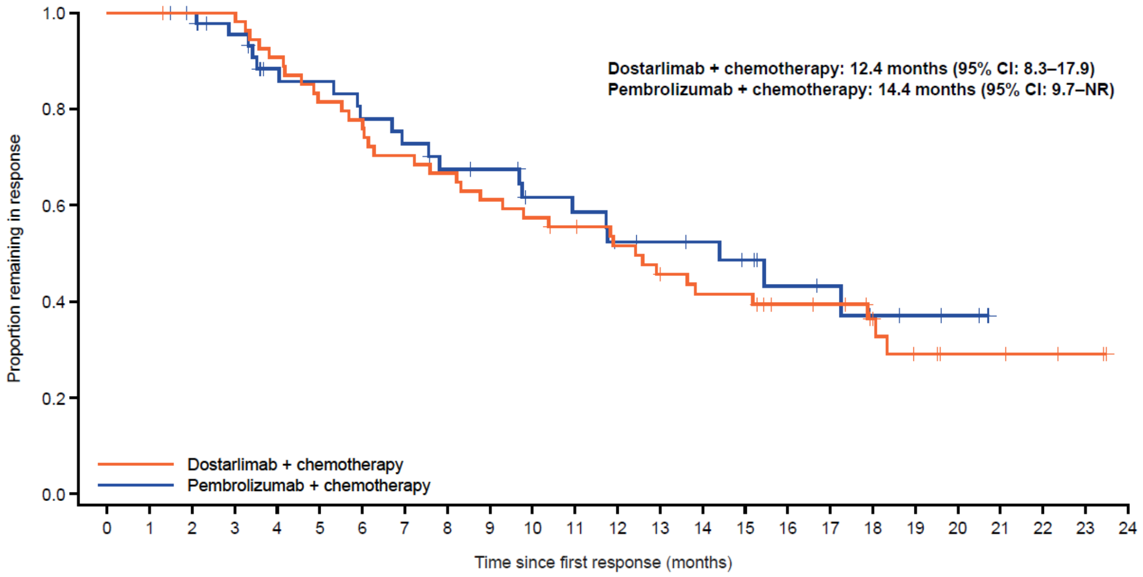

Number at risk
(number of events)

Dostarlimab + chemotherapy	55	55	54	54	49	44	42	38	36	33	31	29	26	23	20	20	16	15	11	7	5	5	2	2	0
	(0)	(0)	(0)	(0)	(5)	(10)	(12)	(16)	(18)	(21)	(23)	(24)	(26)	(29)	(31)	(31)	(32)	(32)	(33)	(35)	(35)	(35)	(35)	(35)	(35)
Pembrolizumab + chemotherapy	48	48	46	41	34	33	30	28	25	24	20	19	16	15	14	12	8	7	5	4	3	0	0	0	0
	(0)	(0)	(0)	(2)	(5)	(6)	(9)	(11)	(13)	(13)	(15)	(16)	(18)	(18)	(18)	(19)	(20)	(20)	(21)	(21)	(21)	(21)	(21)	(21)	(21)

CI, confidence interval; DOR, duration of response; HR, hazard ration; ITT, intention-to-treat; NR, not reached.

Supplementary Figure 2. BICR-assessed maximum percentage reduction from baseline in tumor measurement (ITT population as of August 4, 2022).

A.

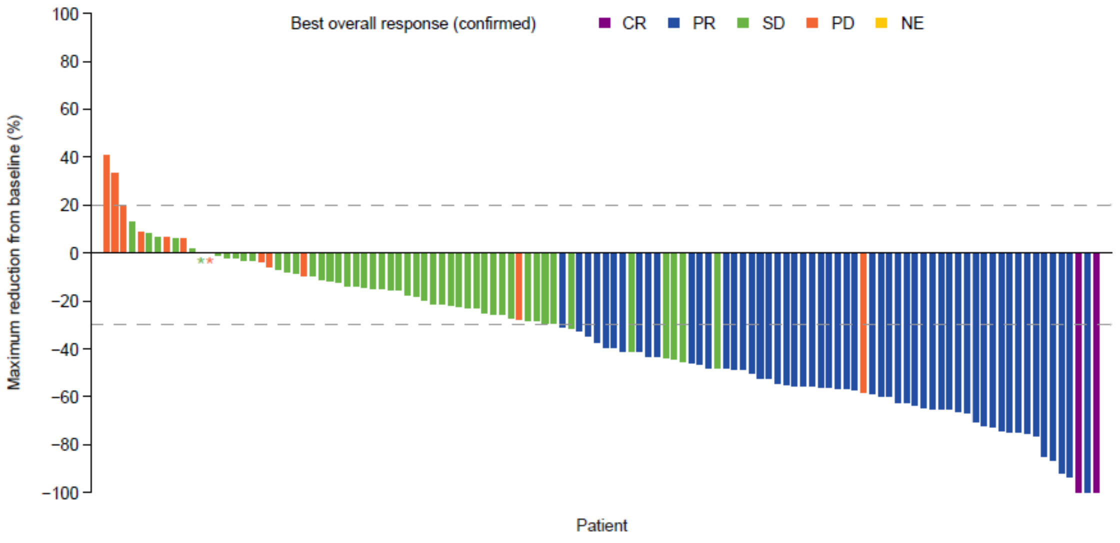

B.

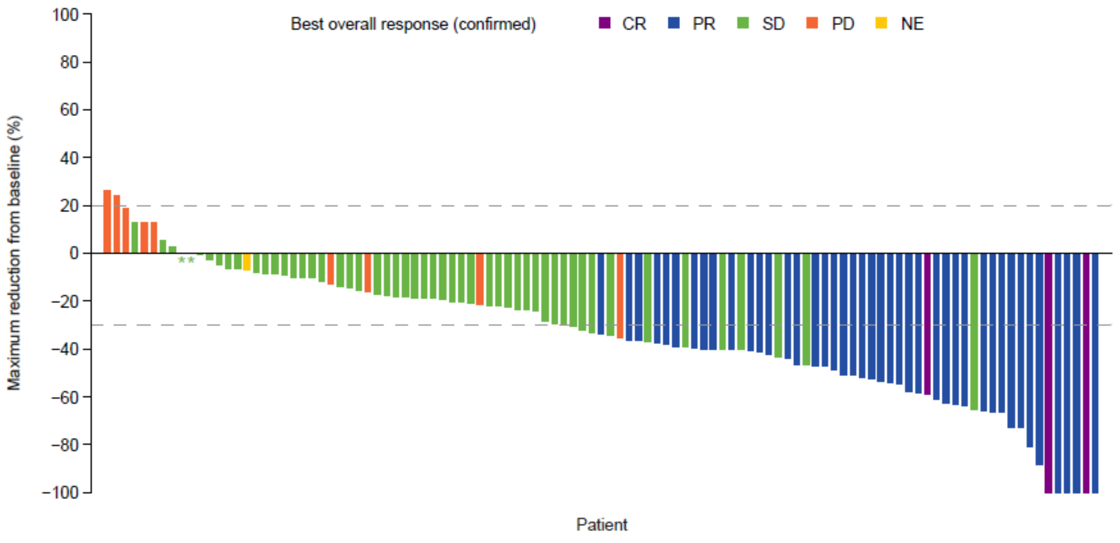

(A) dostarlimab plus chemotherapy (N=121) and (B) pembrolizumab plus chemotherapy (N=122).

*Maximum reduction from baseline of 0%.

Note: one patient with CR with less than 100% BICR-assessed maximum percent reduction from baseline had only lymph node selected by BICR to derive the best overall response as CR.

BICR, blinded independent central review; CR, complete response; ITT, intention-to-treat; NE, not evaluable; PD, progressive disease; PR, partial response; SD, stable disease.

Supplementary Figure 3. Forest plot of differences in ORR (dostarlimab – pembrolizumab, %) for patients receiving dostarlimab + chemotherapy (N=121) and pembrolizumab + chemotherapy (N=122) and 80% CI (ITT population as of August 4, 2022)

Categories	Dostarlimab + chemotherapy n/N (%)	Pembrolizumab + chemotherapy n/N (%)
**Overall**	**56/121 (46)**	**45/122 (37)**
PD-L1 TPS status*		
<1%	14/50 (28)	17/51 (33)
≥1%	42/71 (59)	28/71 (39)
1–49%	22/44 (50)	15/44 (34)
≥50%	20/27 (74)	13/27 (48)
Smoking status*		
Never smoked	7/17 (41)	5/17 (29)
Former/current smoker	49/104 (47)	40/105 (38)
Age group at randomization		
<65 years	30/65 (46)	20/57 (35)
≥65 years	26/56 (46)	25/65 (38)
Sex		
Female	15/36 (42)	15/45 (33)
Male	41/85 (48)	30/77 (39)
Baseline ECOG performance		
0	17/37 (46)	23/50 (46)
1	39/84(46)	22/72 (31)
Brain metastases at baseline		
Yes	11/22 (50)	4/15 (27)
No	45/99 (45)	41/107 (38)
Type of platinum-based chemotherapy		
Cisplatin	9/22 (41)	5/14 (36)
Carboplatin	47/99 (47)	40/108 (37)
Region		
Asia	10/23 (43)	6/21 (29)
Rest of the World	46/98 (47)	39/101 (39)

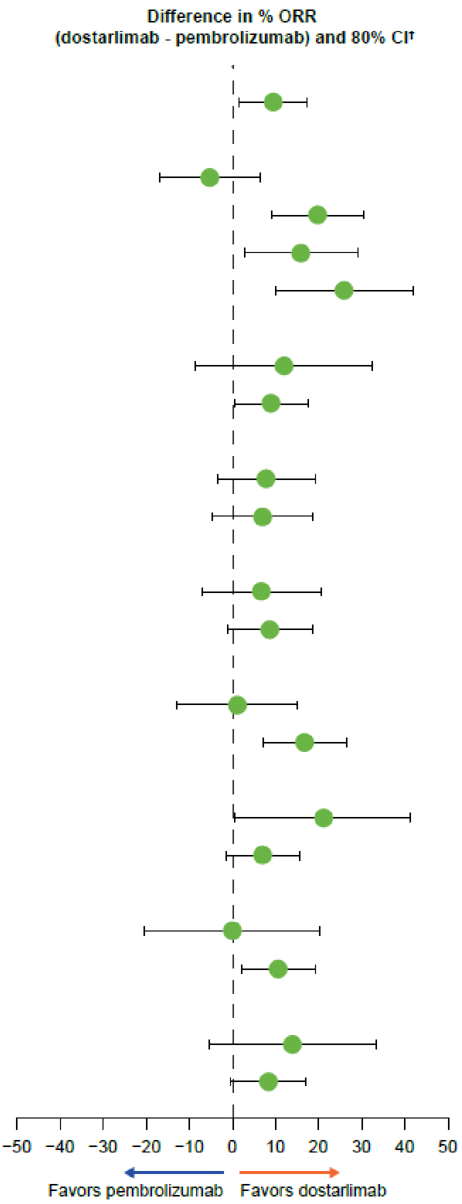

Note: n represents the number of patients in the subgroup with a best overall response of CR or PR and N represents the number of patients in the subgroup.

*Based on the strata collected in Interactive Response Technology at randomization.

†Mantel and Haenszel method with Sato's variance estimator stratified by PD-L1 TPS status (TPS <1% vs 1%–49% vs ≥50%) and smoking status (never vs former/current) based on strata data collected in Interactive Response Technology at randomization.

PD-L1 TPS Status is not included as a stratification factor for the PD-L1 TPS status subgroups. Smoking status is not included as a stratification factor for the smoking status subgroups.

CI, confidence interval; CR, complete response; ECOG, Eastern Cooperative Oncology Group; ORR, overall response rate; PD-L1, programmed death ligand 1; PR, partial response; TPS, tumor proportion score.

Supplementary Figure 4. OS Kaplan–Meier curves by PD-L1 TPS status for dostarlimab plus chemotherapy and pembrolizumab plus chemotherapy (ITT population as of July 7, 2023)

a.

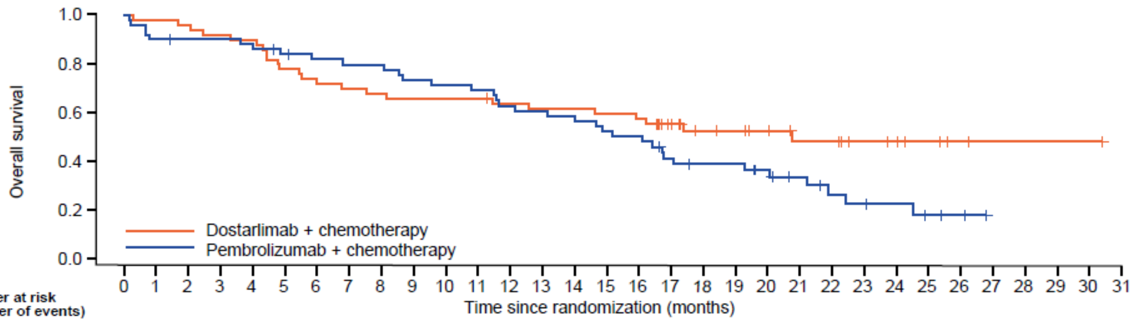

Number at risk (number of events)	0	1	2	3	4	5	6	7	8	9	10	11	12	13	14	15	16	17	18	19	20	21	22	23	24	25	26	27	28	29	30	31
Dostarlimab + chemotherapy	50 (0)	49 (1)	48 (2)	46 (4)	45 (5)	39 (11)	36 (14)	35 (15)	34 (16)	33 (17)	33 (17)	33 (17)	31 (18)	30 (19)	30 (19)	29 (20)	28 (21)	22 (22)	17 (23)	16 (23)	14 (23)	11 (24)	11 (24)	7 (24)	6 (24)	4 (24)	2 (24)	1 (24)	1 (24)	1 (24)	1 (24)	0 (24)
Pembrolizumab + chemotherapy	51 (0)	46 (5)	45 (5)	45 (5)	44 (6)	41 (8)	39 (9)	38 (10)	38 (10)	35 (13)	34 (14)	33 (15)	30 (18)	29 (19)	27 (21)	25 (23)	24 (24)	18 (28)	16 (29)	16 (29)	13 (30)	10 (31)	7 (33)	6 (34)	5 (34)	3 (35)	2 (35)	0 (35)	0 (35)	0 (35)	0 (35)	0 (35)

b.

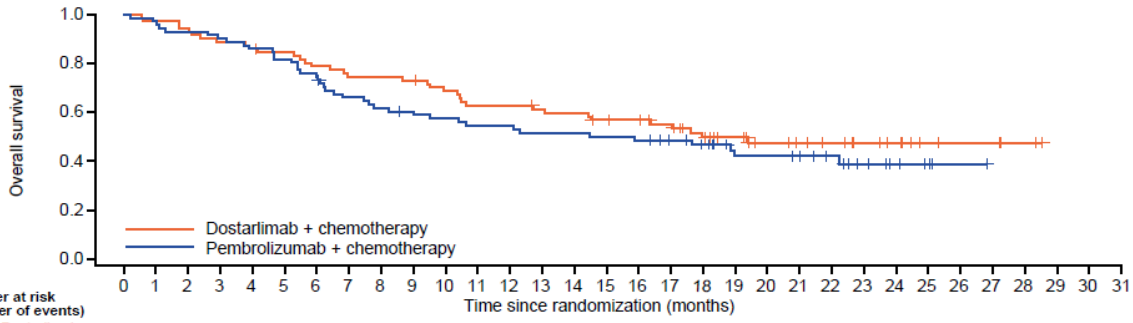

Number at risk (number of events)	0	1	2	3	4	5	6	7	8	9	10	11	12	13	14	15	16	17	18	19	20	21	22	23	24	25	26	27	28	29	30	31
Dostarlimab + chemotherapy	71 (0)	69 (2)	67 (4)	63 (8)	61 (10)	59 (11)	55 (15)	52 (18)	52 (18)	51 (19)	47 (22)	43 (26)	43 (26)	41 (27)	40 (28)	37 (30)	36 (30)	33 (31)	27 (3)	23 (34)	18 (35)	16 (35)	14 (35)	11 (35)	9 (35)	5 (35)	4 (35)	4 (35)	2 (35)	0 (35)	0 (35)	0 (35)
Pembrolizumab + chemotherapy	71 (0)	69 (2)	66 (5)	64 (7)	61 (10)	58 (13)	53 (18)	46 (24)	43 (27)	41 (28)	39 (30)	37 (30)	37 (32)	35 (34)	35 (34)	34 (35)	33 (3)	29 (36)	26 (37)	18 (39)	18 (39)	16 (39)	13 (39)	9 (40)	6 (40)	3 (40)	1 (40)	0 (40)	0 (40)	0 (40)	0 (40)	0 (40)

c.

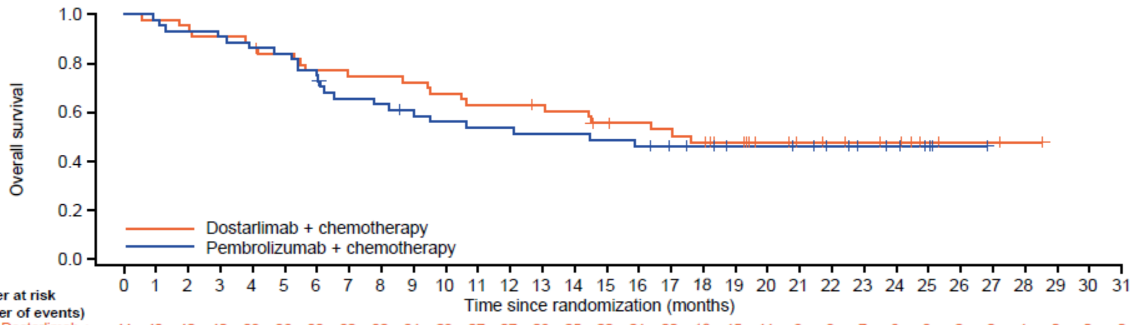

Number at risk (number of events)	0	1	2	3	4	5	6	7	8	9	10	11	12	13	14	15	16	17	18	19	20	21	22	23	24	25	26	27	28	29	30	31
Dostarlimab + chemotherapy	44 (0)	43 (1)	42 (2)	40 (4)	38 (6)	36 (7)	33 (10)	32 (11)	32 (11)	31 (12)	29 (14)	27 (16)	27 (16)	26 (16)	25 (17)	22 (19)	21 (19)	20 (20)	18 (22)	15 (22)	11 (22)	9 (22)	8 (22)	7 (22)	6 (22)	3 (22)	2 (22)	2 (22)	1 (22)	0 (22)	0 (22)	0 (22)
Pembrolizumab + chemotherapy	44 (0)	43 (1)	41 (3)	40 (4)	38 (6)	37 (7)	33 (11)	28 (15)	27 (16)	25 (17)	23 (19)	22 (20)	22 (20)	21 (21)	21 (21)	20 (22)	19 (23)	17 (23)	16 (23)	13 (23)	13 (23)	12 (23)	9 (23)	7 (23)	6 (23)	3 (23)	1 (23)	0 (23)	0 (23)	0 (23)	0 (23)	0 (23)

d.

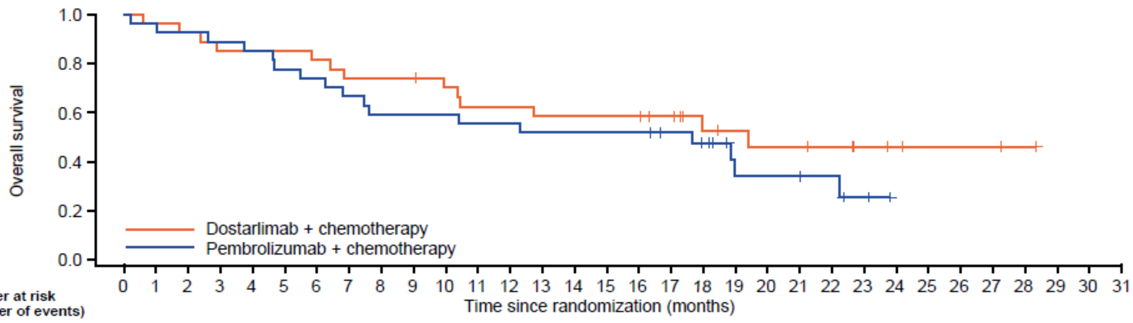

Number at risk (number of events)	0	1	2	3	4	5	6	7	8	9	10	11	12	13	14	15	16	17	18	19	20	21	22	23	24	25	26	27	28	29	30	31
Dostarlimab + chemotherapy	27 (0)	26 (1)	25 (2)	23 (4)	23 (4)	23 (4)	22 (5)	20 (7)	20 (7)	20 (7)	18 (8)	16 (10)	16 (10)	15 (11)	15 (11)	15 (11)	15 (11)	13 (11)	9 (12)	8 (12)	7 (13)	7 (13)	6 (13)	4 (13)	3 (13)	2 (13)	2 (13)	2 (13)	1 (13)	0 (13)	0 (13)	0 (13)
Pembrolizumab + chemotherapy	27 (0)	26 (1)	25 (2)	24 (3)	23 (4)	21 (6)	20 (7)	18 (9)	16 (11)	16 (11)	16 (11)	15 (12)	15 (12)	14 (13)	14 (13)	14 (13)	14 (13)	12 (13)	10 (14)	5 (16)	5 (16)	4 (16)	4 (16)	2 (17)	0 (17)	0 (17)	0 (17)	0 (17)	0 (17)	0 (17)	0 (17)	0 (17)

A) <1% PD-L1 TPS B) ≥1% PD-L1 TPS C) 1–49% PD-L1 TPS and D) ≥50% PD-L1 TPS (data cut-off July 7, 2023).

CI, confidence interval; ITT, intention-to-treat; OS, overall survival; PD-L1, programmed death ligand 1; TPS, tumor proportion score.

Supplementary Figure 5. Tornado plot showing types of AEs in patients receiving dostarlimab + chemotherapy and pembrolizumab + chemotherapy (safety population as of August 4, 2022)

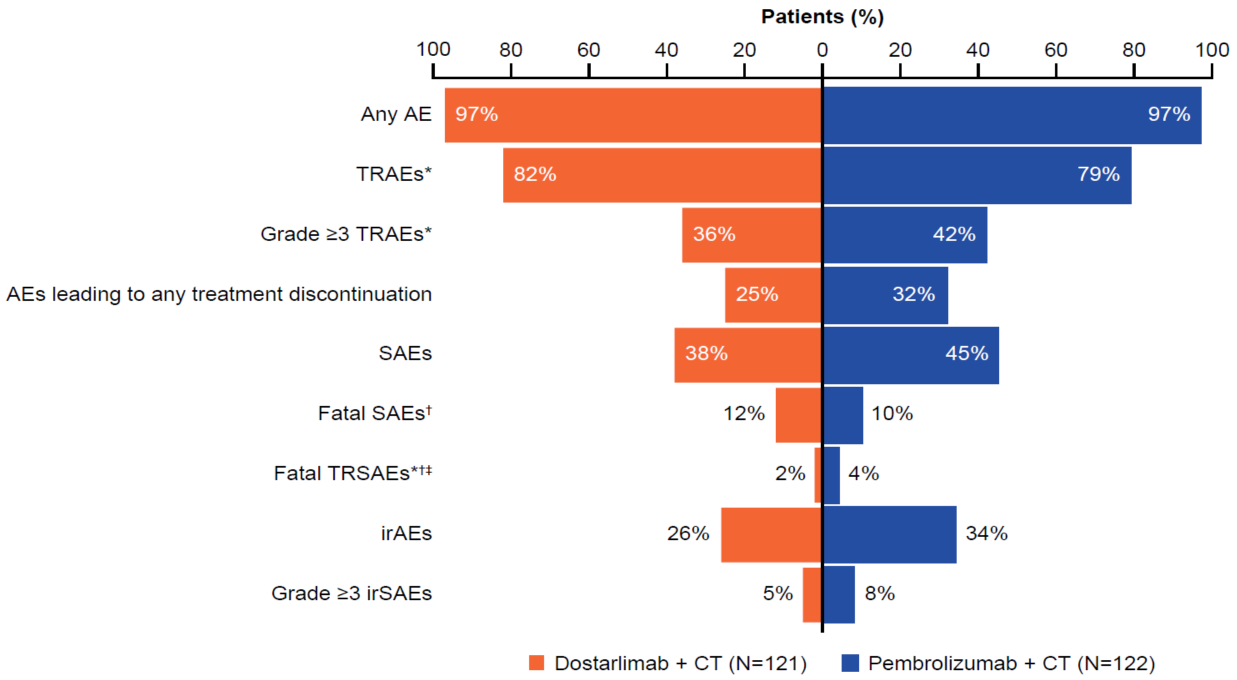

*Adverse events described as treatment-related could be related to any study treatment agent.

†Patients who had a fatal SAE recorded and whose death was not recorded as due unequivocally to disease under study.

‡Fatal TRSAEs for dostarlimab + CT were immune-mediated lung disease, pneumonitis, and urosepsis and for pembrolizumab + CT were myelosuppression, pneumonia, respiratory failure, septic shock, and thrombocytopenia (one patient each).

AE, adverse event; CT, chemotherapy; irAE, immune-related adverse event; irSAE, immune-related serious adverse event; SAE, serious adverse event; TRAE, treatment-related adverse event; TRSAE, treatment-related serious adverse event.
