# Supplementary material for: Dostarlimab or pembrolizumab plus chemotherapy in previously untreated metastatic non-squamous non-small cell lung cancer: the randomized PERLA phase II trial
Source: Nat Commun. 2023 Nov 11;14:7301. doi: 10.1038/s41467-023-42900-4 (PMC10640551; doi:10.1038/s41467-023-42900-4)
Supplement: Supplementary file 3 — Reporting Summary [file 41467_2023_42900_MOESM3_ESM.pdf]

nature portfolio

Corresponding author(s): Sun Min Lim

Last updated by author(s): Oct 6, 2023

Reporting Summary

Nature Portfolio wishes to improve the reproducibility of the work that we publish. This form provides structure for consistency and transparency in reporting. For further information on Nature Portfolio policies, see our Editorial Policies and the Editorial Policy Checklist.

Please do not complete any field with "not applicable" or n/a. Refer to the help text for what text to use if an item is not relevant to your study.
For final submission: please carefully check your responses for accuracy; you will not be able to make changes later.

Statistics

For all statistical analyses, confirm that the following items are present in the figure legend, table legend, main text, or Methods section.

n/a	Confirmed	
☐	☒	The exact sample size (*n*) for each experimental group/condition, given as a discrete number and unit of measurement
☒	☐	A statement on whether measurements were taken from distinct samples or whether the same sample was measured repeatedly
☒	☐	The statistical test(s) used AND whether they are one- or two-sided<br>*Only common tests should be described solely by name; describe more complex techniques in the Methods section.*
☒	☐	A description of all covariates tested
☒	☐	A description of any assumptions or corrections, such as tests of normality and adjustment for multiple comparisons
☐	☒	A full description of the statistical parameters including central tendency (e.g. means) or other basic estimates (e.g. regression coefficient) AND variation (e.g. standard deviation) or associated estimates of uncertainty (e.g. confidence intervals)
☒	☐	For null hypothesis testing, the test statistic (e.g. *F*, *t*, *r*) with confidence intervals, effect sizes, degrees of freedom and *P* value noted<br>*Give P values as exact values whenever suitable.*
☒	☐	For Bayesian analysis, information on the choice of priors and Markov chain Monte Carlo settings
☒	☐	For hierarchical and complex designs, identification of the appropriate level for tests and full reporting of outcomes
☒	☐	Estimates of effect sizes (e.g. Cohen's *d*, Pearson's *r*), indicating how they were calculated

Our web collection on statistics for biologists contains articles on many of the points above.

Software and code

Policy information about availability of computer code

Data collection	Veeva EDC (CDMS) versions 20R3, 21R1, 21R3, 22R1, 22R2 and 22R3 were used for data collection.
Data analysis	SAS software version 9.4 was used for data analysis.

For manuscripts utilizing custom algorithms or software that are central to the research but not yet described in published literature, software must be made available to editors and reviewers. We strongly encourage code deposition in a community repository (e.g. GitHub). See the Nature Portfolio guidelines for submitting code & software for further information.

Data

Policy information about availability of data

All manuscripts must include a data availability statement. This statement should provide the following information, where applicable:

- Accession codes, unique identifiers, or web links for publicly available datasets
- A description of any restrictions on data availability
- For clinical datasets or third party data, please ensure that the statement adheres to our policy

Clinical data are available under restricted access for confidentiality reasons, researchers can request access to our studies by providing a scientific research proposal with a commitment to publish their findings. Researchers whose requests are approved by an independent panel and accepted by GSK are provided access to data in a secure environment upon signing a Data Sharing Agreement (DSA). Review criteria for research proposals are: Scientific rationale and relevance of the proposed research to medical science or patient care, ability of the proposed research plan (design, methods and analysis) to meet the scientific objectives,

qualifications and experience of the research team to conduct the proposed research review, whether the proposal has potential to produce information that may increase the risk of identification of individual research participants, any real or potential conflicts of interest that may impact the planning, conduct or interpretation of the research and proposals to manage these conflicts of interest, and the publication plan for the research. Additionally, patients give permission through an informed consent form to use their data for original studies, so further research must study the medicine or disease that was researched in the original studies. Data will not be provided to requesters where there is a potential conflict of interest, data is to be used for a commercial purpose or there is an actual or potential competitive risk, and researchers are required to sign a DSA, which includes requirements to publish results of the analysis in a scientific journal or pre-print option and open-source release of any software or models. Submitted proposals will be acknowledged within a week and anonymized data will be shared within 30 days of signing the DSA. Access to data and documents is provided for 12 months with the possibility of extension up to an additional 6 months. Please see https://www.gsk-studyregister.com/About_GSK_Patient_Level_Data_Sharing_Final_13July2023.pdf for full details. Access criteria are correct as of August 2023 – the latest information will be available at https://www.gsk-studyregister.com/en/. GSK is committed to share anonymized subject level data from interventional trials as per GSK policies (https://www.gsk.com/en-gb/innovation/trials/data-transparency/) and as applicable. The raw individual participant data are protected and cannot be made publicly available (as source data) due to data privacy laws. The anonymized individual participant data can be requested for further research at https://www.gsk-studyregister.com/en/. The study documents (including the study protocol and statistical analysis plan) are available at https://www.gsk-studyregister.com/en/trial-details/213403. The remaining data are available within the Article and its Supplementary Information.

Human research participants

Policy information about studies involving human research participants and Sex and Gender in Research.

Reporting on sex and gender

Sex was self-reported as part of patient demographics; there were 162 males and 81 females included in this analysis. A prespecified subgroup analysis of overall response rate by sex was conducted and were reported in a poster presented at the European Lung Cancer Congress (ELCC) 2023 in Copenhagen, Denmark (March 29-April 1). Anonymized individual participant data are available on reasonable request (www.clinicalstudydatarequest.com).

Population characteristics

243 patients were enrolled in the study and randomized to either dostarlimab plus chemotherapy (N=121) or pembrolizumab plus chemotherapy (N=122). Median ages were 64.0 and 65.0 years, respectively. 20 patients had Stage I disease at initial diagnosis, 5 had Stage II, 13 had Stage III, 201 had Stage IV, and 4 had unknown Stage. 87 patients had Eastern Cooperative Oncology Group (ECOG) performance status 0 at baseline and 156 patients had ECOG performance status 1. 34 patients had never smoked and 209 were former/current smokers. 238 patients had non-squamous histology and 5 had mixed histology. At baseline, 73 patients had bone metastases, 37 had brain metastases, and 33 had liver metastases. 101 had programmed death-ligand 1 (PD-L1) tumor proportion score (TPS) <1%, 88 had TPS 1–49%, and 54 had TPS ≥50%.

Recruitment

Study eligibility criteria were designed to be as inclusive as possible while maintaining both patient safety and study integrity. This study was double blinded to avoid bias in recruitment. Patients were not compensated for participation in this study, except for reimbursement for travel expenses. Study investigators followed site-specific processes for determining which existing patients met the eligibility requirements for the study and then invited them to review the informed consent form. A total of 352 patients were screened for eligibility. Patients were recruited via competitive enrollment. Following screening, 243 patients at 54 participating sites across 12 countries were enrolled in the study and randomized.

Ethics oversight

All patients provided written informed consent before participation in the study, which was conducted in accordance with the Declaration of Helsinki and International Ethical Guidelines, International Council for Harmonisation Good Clinical Practice guidelines, and all local laws. The study was overseen by an internal safety monitoring committee, comprised of GSK employees who were not part of the dostarlimab development program. The protocol was approved by relevant ethics committees and institutional review boards at each study site:
CIEIS Fabiola (Clinica Universitaria Reina Fabiola),
CEIC Dr. Stamboulian (Instituto de Investigaciones Metabólicas, Centro Polivalente de Asistencia e Inv. Clinica CER, and Centro Oncológico Riojano Integral),
CIEFC- CIE para Ensayos en Farmacología Clínica (Fundacion Respirar),
Comité de Ética en Investigación Fundación OncoSalud (Centro de Investigacion Pergamino)
Comité de Ética en Investigación Clínica (CEIC) (Clinica Viedma S.A.),
Comite de etica en Investigacion CEMER (CEMER),
Instituto de Oncología y Especialidades Médicas (Centro Oncologico de Rosario),
Comité de Ética Instituto Médico Platense (Centro Platense en Investigaciones Respiratorias),
Comitê de Ética em Pesquisa do Instituto Brasileiro de Controle do Câncer (Núcleo de Pesquisa Clínica da Rede São Camilo),
Hospital Univ Cassiano Antônio de Moraes da Univ Federal do Espírito Santo (Hospital Santa Rita),
Comitê de Ética em Pesquisa da Universidade Federal do Caerá (INCA - Instituto Nacional do Cancer),
Comite de Etica em Pesquisa da Universidade do Vale do Taquari – UNIVATES (Sociedade Beneficência e Caridade de Lajeado – Hospital Bruno Born),
Com de Et em Pesq com Seres Humanos da Liga Norte Riograndense Cont o Canc (Liga Norte RioGrandense Contra o Câncer),
Comite de Etica em Pesquisa da Fundacao Pio XII (Fundação PIO XII - Hospital de Câncer de Barretos),
Comité de Ética Científico SSMO (Orlandi Oncologia and Centro de estudios Clinicos SAGA Spa),
Centro de Investigaciones del Cancer James Lind,
CPP Ile-de-France II - Hôpital Necker (CHU de Caen - Hôpital Côte de Nacre, Institut de Cancérologie de l'Ouest - Site Saint Herblain, CHU de Bordeaux - GH Sud - Hôpital Haut-Lévêque, CH Le Mans, Pneumologie – Oncologie Thoracique, CHU de Limoges - CHU Dupuytren 1, and Clinique Teissier – Groupe AHNAC),
Ethikkommission der Aerztekammer Nordrhein (Krankenhaus Merheim),
Ethik-Kommission der Landesärztekammer Hessen (Krankenhaus Nordwest),
Carl von Ossietzky Univerität Oldenburg (Pius Hospital),
St. Vincentius-Kliniken gAG Karlsruhe),
Ethikkommission des Landes Berlin (Charite-Universitaetsmedizin Berlin),
Ethik-Kommission der Landesärztekammer Hessen (Fachklinik f. Lungenerkrankungen and Klinikum Kassel GmbH),
Ethik-Kommission der Bayerischen Landesärztekammer (Studienzentrum Haematologie/Onkologie/Diabetologie),
Segreteria Scientifico-Amministrativa (Arnas Garibaldi - P.O. Nesima, Dipartimento Oncologico U.O.C. Oncologia medica),

Comitato Etico Regionale Unico c/o AOU Santa Maria della Misericordia (Centro Di Riferimento Oncologico - IRCCS - Sevizio Sanitario Regionale FVG)
Com.Etico IRCCS – Ist. Europeo di Oncologia e Centro Cardiologico Monzino (Istituto Europeo di Oncologia),
Comitato Etico di Brescia, Segreteria Scientifico-Amministrativa (Spedali Civili di Brescia),
Comitato Etico Ind dell'Istutituto Naz per lo Studio e la Cura dei Tumori (Istituto Nazionale dei Tumori IRCCS).
Comitato Etico Lazio 1, Segreteria Scientifico-Amministrativa (Azienda Ospedaliera San Camillo Forlanini),
Comitato Etico dell'A.O.R.N. dei Colli di Napoli (A.O.R.N. Ospedali dei Colli "Monaldi-Cotugno-CTO),
Samsung Medical Center,
Asan Medical Center,
Severance Hospital,
Chungbuk National University Hospital,
Inje University Haeundae Paik,
Kom Bioet przy Okręgowej Izbie Lekarskiej Wielkopolskiej Izby Lekarskiej (Centrum Medyczne Pratia, Samodzielny Publiczny Zespol Gruzlicy i Chorob Pluc, Samodzielny Publiczny Szpital Kliniczny w Lublinie, Klinika Pneumonologii, Ars Medical Sp. z o.o, ul., Centrum Terapii Wspolczesnej, Centrum Onkologii im. prof. F. Lukaszczyka and Med-Polonia Sp. z o. o. NSZOZ),
Comisia Nationala de Bioetica a medicamentelor si a Dispozitivelor Medicale (SC Radiotherapy Center Cluj SRL),
National Ethics committee (Spitalul de Psihiatrie Titan Dr Constantin Gorgos),
Comisia Nationala de Bioetica a medicamentelor si a Dispozitivelor Medicale (Centrul de Oncologie "Sf. Nectarie"),
Hospital del Mar (Hospital del Mar Medical Oncology Department, Complejo Hospitario Nuestra, Complejo Hospitalario de Jaén, Hospital Universitario Virgen de la Victoria, and Hospital Universitario Lucus Augusti),
Tri-Service General Hospital,
Changhua Christian Hospital,
United States Oncology Incorporated Institutional Review Board (The Texas Cancer Center, Rocky Mountain Cancer Centers-Sky Ridge, Cancer Care Centers of Brevard, Virginia Cancer Specialists, Woodlands Medical Specialists, Texas Oncology - Tyler, Oncology Hematology Care).

Note that full information on the approval of the study protocol must also be provided in the manuscript.

Field-specific reporting

Please select the one below that is the best fit for your research. If you are not sure, read the appropriate sections before making your selection.

☒ Life sciences ☐ Behavioural & social sciences ☐ Ecological, evolutionary & environmental sciences

For a reference copy of the document with all sections, see nature.com/documents/nr-reporting-summary-flat.pdf

Life sciences study design

All studies must disclose on these points even when the disclosure is negative.

Sample size	The planned sample size for the study was 240 (approximately 120 patients in each arm), providing 85% power to detect a 15% difference in ORR between treatment groups (with a 10% one-sided type I error rate) if the true ORR was 45% for both treatment groups.
Data exclusions	No data was excluded.
Replication	This large clinical trial was sufficiently powered to evaluate the study hypothesis, with 243 patients in the intent-to-treat population. This study was conducted across 54 study sites in 12 countries. Due to the nature of the study, no replication of experiments was performed.
Randomization	Eligible patients were randomized 1:1 via blocking using an interactive web response system (RAMOS NG), to receive chemotherapy in combination with either dostarlimab or pembrolizumab. Randomization was stratified by PD-L1 status (TPS <1% vs 1–49% vs ≥50%) and smoking status (never vs former/current).
Blinding	Patients, relevant study staff, and investigators were blinded to study treatment; details of blinding were documented in site-specific blinding plans.

Behavioural & social sciences study design

All studies must disclose on these points even when the disclosure is negative.

Study description	*Briefly describe the study type including whether data are quantitative, qualitative, or mixed-methods (e.g. qualitative cross-sectional, quantitative experimental, mixed-methods case study).*
Research sample	*State the research sample (e.g. Harvard university undergraduates, villagers in rural India) and provide relevant demographic information (e.g. age, sex) and indicate whether the sample is representative. Provide a rationale for the study sample chosen. For studies involving existing datasets, please describe the dataset and source.*
Sampling strategy	*Describe the sampling procedure (e.g. random, snowball, stratified, convenience). Describe the statistical methods that were used to predetermine sample size OR if no sample-size calculation was performed, describe how sample sizes were chosen and provide a*

	rationale for why these sample sizes are sufficient. For qualitative data, please indicate whether data saturation was considered, and what criteria were used to decide that no further sampling was needed.
Data collection	*Provide details about the data collection procedure, including the instruments or devices used to record the data (e.g. pen and paper, computer, eye tracker, video or audio equipment) whether anyone was present besides the participant(s) and the researcher, and whether the researcher was blind to experimental condition and/or the study hypothesis during data collection.*
Timing	*Indicate the start and stop dates of data collection. If there is a gap between collection periods, state the dates for each sample cohort.*
Data exclusions	*If no data were excluded from the analyses, state so OR if data were excluded, provide the exact number of exclusions and the rationale behind them, indicating whether exclusion criteria were pre-established.*
Non-participation	*State how many participants dropped out/declined participation and the reason(s) given OR provide response rate OR state that no participants dropped out/declined participation.*
Randomization	*If participants were not allocated into experimental groups, state so OR describe how participants were allocated to groups, and if allocation was not random, describe how covariates were controlled.*

Ecological, evolutionary & environmental sciences study design

All studies must disclose on these points even when the disclosure is negative.

Study description	*Briefly describe the study. For quantitative data include treatment factors and interactions, design structure (e.g. factorial, nested, hierarchical), nature and number of experimental units and replicates.*
Research sample	*Describe the research sample (e.g. a group of tagged Passer domesticus, all Stenocereus thurberi within Organ Pipe Cactus National Monument), and provide a rationale for the sample choice. When relevant, describe the organism taxa, source, sex, age range and any manipulations. State what population the sample is meant to represent when applicable. For studies involving existing datasets, describe the data and its source.*
Sampling strategy	*Note the sampling procedure. Describe the statistical methods that were used to predetermine sample size OR if no sample-size calculation was performed, describe how sample sizes were chosen and provide a rationale for why these sample sizes are sufficient.*
Data collection	*Describe the data collection procedure, including who recorded the data and how.*
Timing and spatial scale	*Indicate the start and stop dates of data collection, noting the frequency and periodicity of sampling and providing a rationale for these choices. If there is a gap between collection periods, state the dates for each sample cohort. Specify the spatial scale from which the data are taken*
Data exclusions	*If no data were excluded from the analyses, state so OR if data were excluded, describe the exclusions and the rationale behind them, indicating whether exclusion criteria were pre-established.*
Reproducibility	*Describe the measures taken to verify the reproducibility of experimental findings. For each experiment, note whether any attempts to repeat the experiment failed OR state that all attempts to repeat the experiment were successful.*
Randomization	*Describe how samples/organisms/participants were allocated into groups. If allocation was not random, describe how covariates were controlled. If this is not relevant to your study, explain why.*
Blinding	*Describe the extent of blinding used during data acquisition and analysis. If blinding was not possible, describe why OR explain why blinding was not relevant to your study.*

Did the study involve field work? ☐ Yes ☐ No

Field work, collection and transport

Field conditions	*Describe the study conditions for field work, providing relevant parameters (e.g. temperature, rainfall).*
Location	*State the location of the sampling or experiment, providing relevant parameters (e.g. latitude and longitude, elevation, water depth).*
Access & import/export	*Describe the efforts you have made to access habitats and to collect and import/export your samples in a responsible manner and in compliance with local, national and international laws, noting any permits that were obtained (give the name of the issuing authority, the date of issue, and any identifying information).*
Disturbance	*Describe any disturbance caused by the study and how it was minimized.*

Reporting for specific materials, systems and methods

We require information from authors about some types of materials, experimental systems and methods used in many studies. Here, indicate whether each material, system or method listed is relevant to your study. If you are not sure if a list item applies to your research, read the appropriate section before selecting a response.

Materials & experimental systems

n/a	Involved in the study
☒	☐ Antibodies
☒	☐ Eukaryotic cell lines
☒	☐ Palaeontology and archaeology
☒	☐ Animals and other organisms
☐	☒ Clinical data
☒	☐ Dual use research of concern

Methods

n/a	Involved in the study
☒	☐ ChIP-seq
☒	☐ Flow cytometry
☒	☐ MRI-based neuroimaging

Antibodies

Antibodies used	*Describe all antibodies used in the study; as applicable, provide supplier name, catalog number, clone name, and lot number.*
Validation	*Describe the validation of each primary antibody for the species and application, noting any validation statements on the manufacturer's website, relevant citations, antibody profiles in online databases, or data provided in the manuscript.*

Eukaryotic cell lines

Policy information about cell lines and Sex and Gender in Research

Cell line source(s)	*State the source of each cell line used and the sex of all primary cell lines and cells derived from human participants or vertebrate models.*
Authentication	*Describe the authentication procedures for each cell line used OR declare that none of the cell lines used were authenticated.*
Mycoplasma contamination	*Confirm that all cell lines tested negative for mycoplasma contamination OR describe the results of the testing for mycoplasma contamination OR declare that the cell lines were not tested for mycoplasma contamination.*
Commonly misidentified lines (See ICLAC register)	*Name any commonly misidentified cell lines used in the study and provide a rationale for their use.*

Palaeontology and Archaeology

Specimen provenance	*Provide provenance information for specimens and describe permits that were obtained for the work (including the name of the issuing authority, the date of issue, and any identifying information). Permits should encompass collection and, where applicable, export.*
Specimen deposition	*Indicate where the specimens have been deposited to permit free access by other researchers.*
Dating methods	*If new dates are provided, describe how they were obtained (e.g. collection, storage, sample pretreatment and measurement), where they were obtained (i.e. lab name), the calibration program and the protocol for quality assurance OR state that no new dates are provided.*

☐ Tick this box to confirm that the raw and calibrated dates are available in the paper or in Supplementary Information.

Ethics oversight	*Identify the organization(s) that approved or provided guidance on the study protocol, OR state that no ethical approval or guidance was required and explain why not.*

Note that full information on the approval of the study protocol must also be provided in the manuscript.

Animals and other research organisms

Policy information about studies involving animals; ARRIVE guidelines recommended for reporting animal research, and Sex and Gender in Research

Laboratory animals	*For laboratory animals, report species, strain and age OR state that the study did not involve laboratory animals.*

Wild animals

Provide details on animals observed in or captured in the field; report species and age where possible. Describe how animals were caught and transported and what happened to captive animals after the study (if killed, explain why and describe method; if released, say where and when) OR state that the study did not involve wild animals.

Reporting on sex

Indicate if findings apply to only one sex; describe whether sex was considered in study design, methods used for assigning sex. Provide data disaggregated for sex where this information has been collected in the source data as appropriate; provide overall numbers in this Reporting Summary. Please state if this information has not been collected. Report sex-based analyses where performed, justify reasons for lack of sex-based analysis.

Field-collected samples

For laboratory work with field-collected samples, describe all relevant parameters such as housing, maintenance, temperature, photoperiod and end-of-experiment protocol OR state that the study did not involve samples collected from the field.

Ethics oversight

Identify the organization(s) that approved or provided guidance on the study protocol, OR state that no ethical approval or guidance was required and explain why not.

Note that full information on the approval of the study protocol must also be provided in the manuscript.

Clinical data

Policy information about clinical studies

All manuscripts should comply with the ICMJE guidelines for publication of clinical research and a completed CONSORT checklist must be included with all submissions.

Clinical trial registration

NCT04581824

Study protocol

Study documents can be requested for further research (www.clinicalstudydatarequest.com).

Data collection

Data were collected at individual participating study sites by study site staff using an electronic case report form (eCRF). Patients were enrolled from 54 sites across 12 countries (Republic of Korea, Taiwan, France, Germany, Italy, Poland, Romania, Spain, Argentina, Brazil, Chile, and the US) between November 19, 2020 and February 18, 2022. Data cut offs for the results described in this paper was August 4, 2022 and July 7, 2023. Data collection took place from enrollment of the first patient to data cut-off for both data cut offs.

Outcomes

The primary endpoint was confirmed ORR, as measured by BICR per RECIST v1.1 criteria, defined as the proportion of patients with a best overall response of either CR or PR. ORR was assessed after all patients had completed the third on-study tumor assessment (after approximately 6 months) or had discontinued from the study, whichever occurred first. Patients with unknown or missing responses were counted as 'not done' but were included in the denominator when calculating percentage of responses.
Secondary efficacy endpoints included PFS (defined as the time from date of randomization to date of disease progression [as per RECIST v1.1 by Investigator assessment] or any-cause death, whichever occurred first), DOR (defined as the time from first documented CR or PR until documented disease progression [as per RECIST v1.1 by BICR], or death, whichever occurs first) and OS (defined as the time from date of randomization to the date of any-cause death). Prespecified analyses of ORR, PFS and OS by PD-L1 subgroups TPS <1%, TPS 1–49%, TPS ≥50%, and TPS ≥1% were also performed.
Safety assessments included vital signs, clinical laboratory parameters, and incidence of TEAEs, SAEs, TEAEs leading to death, and AEs leading to discontinuation. AEs were coded using standard Medical Dictionary for Regulatory Activities (MedDRA) terminology and graded by the Investigator according to the National Cancer Institute Common Terminology Criteria for Adverse Events (NCI-CTCAE) v5.0.
Primary analyses of ORR, PFS and safety were preformed per August 4, 2022 data cut off. Due to the immature OS and DOR data from the August 4, 2022 data cut, additional analyses were planned for more mature data. Updated analyses of ORR, duration of response (DOR), overall survival (OS) and safety data were also preformed per July 7, 2023 data cut-off.

Dual use research of concern

Policy information about dual use research of concern

Hazards

Could the accidental, deliberate or reckless misuse of agents or technologies generated in the work, or the application of information presented in the manuscript, pose a threat to:

No	Yes	
☐	☐	Public health
☐	☐	National security
☐	☐	Crops and/or livestock
☐	☐	Ecosystems
☐	☐	Any other significant area

Experiments of concern

Does the work involve any of these experiments of concern:

No	Yes	
☐	☐	Demonstrate how to render a vaccine ineffective
☐	☐	Confer resistance to therapeutically useful antibiotics or antiviral agents
☐	☐	Enhance the virulence of a pathogen or render a nonpathogen virulent
☐	☐	Increase transmissibility of a pathogen
☐	☐	Alter the host range of a pathogen
☐	☐	Enable evasion of diagnostic/detection modalities
☐	☐	Enable the weaponization of a biological agent or toxin
☐	☐	Any other potentially harmful combination of experiments and agents

ChIP-seq

Data deposition

☐ Confirm that both raw and final processed data have been deposited in a public database such as GEO.

☐ Confirm that you have deposited or provided access to graph files (e.g. BED files) for the called peaks.

Data access links<br>*May remain private before publication.*	*For "Initial submission" or "Revised version" documents, provide reviewer access links. For your "Final submission" document, provide a link to the deposited data.*
Files in database submission	*Provide a list of all files available in the database submission.*
Genome browser session<br>(e.g. UCSC)	*Provide a link to an anonymized genome browser session for "Initial submission" and "Revised version" documents only, to enable peer review. Write "no longer applicable" for "Final submission" documents.*

Methodology

Replicates	*Describe the experimental replicates, specifying number, type and replicate agreement.*
Sequencing depth	*Describe the sequencing depth for each experiment, providing the total number of reads, uniquely mapped reads, length of reads and whether they were paired- or single-end.*
Antibodies	*Describe the antibodies used for the ChIP-seq experiments; as applicable, provide supplier name, catalog number, clone name, and lot number.*
Peak calling parameters	*Specify the command line program and parameters used for read mapping and peak calling, including the ChIP, control and index files used.*
Data quality	*Describe the methods used to ensure data quality in full detail, including how many peaks are at FDR 5% and above 5-fold enrichment.*
Software	*Describe the software used to collect and analyze the ChIP-seq data. For custom code that has been deposited into a community repository, provide accession details.*

Flow Cytometry

Plots

Confirm that:

☐ The axis labels state the marker and fluorochrome used (e.g. CD4-FITC).

☐ The axis scales are clearly visible. Include numbers along axes only for bottom left plot of group (a 'group' is an analysis of identical markers).

☐ All plots are contour plots with outliers or pseudocolor plots.

☐ A numerical value for number of cells or percentage (with statistics) is provided.

Methodology

Sample preparation	*Describe the sample preparation, detailing the biological source of the cells and any tissue processing steps used.*
Instrument	*Identify the instrument used for data collection, specifying make and model number.*

Software | *Describe the software used to collect and analyze the flow cytometry data. For custom code that has been deposited into a community repository, provide accession details.*

Cell population abundance | *Describe the abundance of the relevant cell populations within post-sort fractions, providing details on the purity of the samples and how it was determined.*

Gating strategy | *Describe the gating strategy used for all relevant experiments, specifying the preliminary FSC/SSC gates of the starting cell population, indicating where boundaries between "positive" and "negative" staining cell populations are defined.*

☐ Tick this box to confirm that a figure exemplifying the gating strategy is provided in the Supplementary Information.

Magnetic resonance imaging

Experimental design

Design type | *Indicate task or resting state; event-related or block design.*

Design specifications | *Specify the number of blocks, trials or experimental units per session and/or subject, and specify the length of each trial or block (if trials are blocked) and interval between trials.*

Behavioral performance measures | *State number and/or type of variables recorded (e.g. correct button press, response time) and what statistics were used to establish that the subjects were performing the task as expected (e.g. mean, range, and/or standard deviation across subjects).*

Acquisition

Imaging type(s) | *Specify: functional, structural, diffusion, perfusion.*

Field strength | *Specify in Tesla*

Sequence & imaging parameters | *Specify the pulse sequence type (gradient echo, spin echo, etc.), imaging type (EPI, spiral, etc.), field of view, matrix size, slice thickness, orientation and TE/TR/flip angle.*

Area of acquisition | *State whether a whole brain scan was used OR define the area of acquisition, describing how the region was determined.*

Diffusion MRI ☐ Used ☐ Not used

Preprocessing

Preprocessing software | *Provide detail on software version and revision number and on specific parameters (model/functions, brain extraction, segmentation, smoothing kernel size, etc.).*

Normalization | *If data were normalized/standardized, describe the approach(es): specify linear or non-linear and define image types used for transformation OR indicate that data were not normalized and explain rationale for lack of normalization.*

Normalization template | *Describe the template used for normalization/transformation, specifying subject space or group standardized space (e.g. original Talairach, MNI305, ICBM152) OR indicate that the data were not normalized.*

Noise and artifact removal | *Describe your procedure(s) for artifact and structured noise removal, specifying motion parameters, tissue signals and physiological signals (heart rate, respiration).*

Volume censoring | *Define your software and/or method and criteria for volume censoring, and state the extent of such censoring.*

Statistical modeling & inference

Model type and settings | *Specify type (mass univariate, multivariate, RSA, predictive, etc.) and describe essential details of the model at the first and second levels (e.g. fixed, random or mixed effects; drift or auto-correlation).*

Effect(s) tested | *Define precise effect in terms of the task or stimulus conditions instead of psychological concepts and indicate whether ANOVA or factorial designs were used.*

Specify type of analysis: ☐ Whole brain ☐ ROI-based ☐ Both

Statistic type for inference
(See Eklund et al. 2016) | *Specify voxel-wise or cluster-wise and report all relevant parameters for cluster-wise methods.*

Correction | *Describe the type of correction and how it is obtained for multiple comparisons (e.g. FWE, FDR, permutation or Monte Carlo).*

Models & analysis

n/a	Involved in the study
☐	☐ Functional and/or effective connectivity
☐	☐ Graph analysis
☐	☐ Multivariate modeling or predictive analysis

Functional and/or effective connectivity

Report the measures of dependence used and the model details (e.g. Pearson correlation, partial correlation, mutual information).

Graph analysis

Report the dependent variable and connectivity measure, specifying weighted graph or binarized graph, subject- or group-level, and the global and/or node summaries used (e.g. clustering coefficient, efficiency, etc.).

Multivariate modeling and predictive analysis

Specify independent variables, features extraction and dimension reduction, model, training and evaluation metrics.

This checklist template is licensed under a Creative Commons Attribution 4.0 International License, which permits use, sharing, adaptation, distribution and reproduction in any medium or format, as long as you give appropriate credit to the original author(s) and the source, provide a link to the Creative Commons license, and indicate if changes were made. The images or other third party material in this article are included in the article's Creative Commons license, unless indicated otherwise in a credit line to the material. If material is not included in the article's Creative Commons license and your intended use is not permitted by statutory regulation or exceeds the permitted use, you will need to obtain permission directly from the copyright holder. To view a copy of this license, visit http://creativecommons.org/licenses/by/4.0/
